# Application of medical cannabis in unstable angina and coronary artery disease: A case report

**DOI:** 10.1097/MD.0000000000025172

**Published:** 2021-03-19

**Authors:** Brian L. Shaffer, Garrison M. Davis, Marc A. Incitti, Brian J. Piper, Brian V. Entler

**Affiliations:** a Center for Primary Care Medicine, Lawrenceville, NJ; b Geisinger Commonwealth School of Medicine, Scranton; c Center for Pharmacy Innovation and Outcomes, Forty Fort, PA.

**Keywords:** cannabidiol, chronic pain, marijuana, medicinal cannabis, tetrahydrocannabinol

## Abstract

**Rationale:**

First discovered in 1990, the endocannabinoid system (ECS) was initially shown to have an intimate relationship with central areas of the nervous system associated with pain, reward, and motivation. Recently, however, the ECS has been extensively implicated in the cardiovascular system with contractility, heart rate, blood pressure, and vasodilation. Emerging data demonstrate modulation of the ECS plays an essential role in cardio metabolic risk, atherosclerosis, and can even limit damage to cardiomyocytes during ischemic events.

**Patient Concerns:**

This case describes a 63-year-old man who presented to a primary care physician for a medical cannabis (MC) consult due to unstable angina (UA) not relieved by morphine or cardiac medications; having failed all first- and second-line polypharmaceutical therapies. The patient reported frequent, unprovoked, angina and exertional dyspnea.

**Diagnosis:**

Having a complex cardiac history, the patient first presented 22 years ago after a suspected myocardial infarction. He re-presented in 2010 and underwent stent placement at that time for inoperable triple-vessel coronary artery disease (CAD) which was identified via percutaneous transluminal coronary angioplasty. UA developed on follow-up and, despite medical management over the past 6 years, became progressively debilitating.

**Interventions and Outcomes:**

In conjunction with his standard cardiac care, patient had a gradual lessening of UA-related pain, including frequency and character, after using an edible form of MC (1:1 cannabidiol:Δ^9^-tetrahydrocannabinol). Following continued treatment, he ceased long-term morphine treatment and described the pain as no longer crippling. As demonstrated by his exercise tolerance tests, the patient experienced an improved functional capacity and reported an increase in his daily functioning, and overall activity.

**Lessons:**

This case uniquely highlights MC in possibly reducing the character, quality, and frequency of UA, whereas concordantly improving functional cardiac capacity in a patient with CAD. Additional case reports are necessary to verify this.

## 1 Introduction

Upon consumption, cannabis rapidly stimulates the endocannabinoid system (ECS) with an onset 20 minutes to 2 hours after ingestion or from 2 to 20 minutes after inhalation. The ECS consists of the neurotransmitters anandamide and 2-arachidonoylglycerol, the CB_1_ and CB_2_ receptor system, as well as other noncannabinoid targets (g-protein coupled receptor [GPCRs]).^[1]^ The CB_1_ receptor is most concentrated in the hippocampus, periaqueductal grey, spinal trigeminal nucleus, amygdala, cerebellum, basal ganglia,^[2]^ and cardiomyocytes where they promote negative inotrope and vasodilation.^[3,4]^ The CB_2_ receptor system is largely concentrated outside of the central nervous system and primarily exhibits its effects on the immune system, systemic inflammatory state, the gut, cardiomyocytes, and vascular smooth muscle.^[3]^

The physiological response to ingestion or inhalation of cannabis in an acute setting is well defined and consists of hypertension and reflex tachycardia.^[3,4]^ Whereas, in long-term users, or under chronic administration, cannabis produces bradycardia and hypotension.^[4]^ It is largely believed that these physiological effects are controlled by a fluctuating excitation/inhibition of CB_1_ receptors on postsynaptic sympathetic and parasympathetic fibers, during acute and chronic administration, respectively.^[2,4]^ Cannabis has also been shown to have significant properties as a vasodilator, acting through noncannabinoid targets, such as the newly discovered GPCRs class known as the transient receptor potential cation channel subfamily V member 1.^[1]^ These effects are seen within minutes to hours of administration and mediated by the ECS; playing an essential role in cardio metabolic risk, atherosclerosis, and limiting damage to cardiomyocytes during ischemic events.^[3]^

## 2 Case presentation

This case describes a 63-year-old man, former narcotics officer, with a complex cardiac history who first presented 22 years ago after a suspected myocardial infarction (MI). He has a 21-pack year history of smoking, but stopped immediately after his MI. In 2009, the patient re-presented to cardiology and underwent a nuclear stress test demonstrating moderate ischemia in the anterior and anterolateral walls. A subsequent angiogram showed significant calcification in the left main coronary artery (LMCA) and the proximal left anterior descending artery (LAD). Disease in the right main coronary artery (RMCA) was also noted. He was referred for a 2D echocardiogram (ECHO) and recatheterization to explore the extent of his coronary artery disease (CAD; see supplemental Table 1, summary of cardiac history [2009–2010] and see supplemental Table 3, which illustrates both the single-photon emission computerized tomography and exercise tolerance test [ETT] findings [2010–2018]).

Stents were placed for diffuse atherosclerotic changes (see supplemental Table 1, summary of cardiac history [2009–2010]), and inoperable triple-vessel CAD which was identified via percutaneous transluminal coronary angioplasty. The RMCA was small, nondominant, with subtotal occlusion. There was 10% luminal compromise of the LMCA. Heavy calcification was noted within all of the diagonals of the LAD, whereas the LAD itself was diffusely diseased with areas of ectatic dilation and moderate stenosis, 60% to 70%. There was an aneurysmal dilation in the proximal LAD and a bare-metal stent was deployed distal to the dilation, in the mid-LAD. The left circumflex (LCX) was the dominant vessel and the AV segment was diffusely dilated and ectatic. The first and second obtuse marginal (OM1 and OM2) were 100% occluded and were noted to fill retrograde. A cutting balloon was unable to cross the area of disease in the both the OM1 and OM2. The obtuse marginal 3 (OM3) had multiple stenotic regions with the worst being 90% occluded and a bare-metal stent was deployed at the location of the near-total occlusion. The posterior descending artery was large and also diffusely atherosclerotic with multiple angiographic stenotic areas of varying severity; the worst located in the distal segments. During follow-up he reported little relief from the stent(s) and presented with angina for the first time.

An ECHO in March of 2010 showed a large wall-motion abnormality in the lateral and inferolateral walls. A low-normal left ventricular ejection fraction (LVEF), 40% to 50%, was also noted despite first-line mediations, which were modified for maximal impact and failing. Medications at that time where aspirin 325 mg, clopidogrel 75 mg, atorvastatin 40 mg, niacin 500 mg, omega-3, and carvedilol 3.125 mg (see table supplemental 2, for all medications and dosages [2009–2019]). The atorvastatin was subsequently terminated and rosuvastatin 20 mg was initiated, as was lisinopril 25 mg. The carvedilol was also increased to 6.25 mg (1.5 tabs, daily). These changes in his pharmacologic treatment produced significant results in his functional capacity, as demonstrated by his LVEF of 60% during a cardiac cath later in April 2010.

The patient underwent a cardiac recatheterization in April 2010 to visualize his coronary anatomy, assess the patency of the previous stents, and to place drug-eluting stents in other areas of significant disease, if necessary. The left heart catheterization demonstrated an RMCA with diffuse disease and total distal occlusion, LMCA with 40% occlusion, and an LCX with a fully occluded diagonal OM1 with a patent bare-metal stent in the proximal-OM3. The bare-metal stent in the mid-LAD was also patent. The LAD-1, however, was functionally occluded with poor run-off; it is noted that the vessel originally supplied a large area of myocardium. Drug-eluting stents were not placed, and the patient continued to report significant episodes of pain and discomfort consistent with stable angina.

In September 2010, he had his first ETT to assess performance and to quantify his ischemia (see supplemental Table 3, which illustrates both the single-photon emission computerized tomography and ETT findings [2010–2018]). The electrocardiogram (EKG) showed 2 mm ST depressions, exercise-induced premature ventricualar contractions, and the test was abandoned.

In 2011, the patient underwent a Single-photon emission computed tomography (SPECT) study. Using a modified Bruce protocol, he completed 2 minutes of stage III until he began to experience significant pain. The patient chose to continue the test using regadenoson to ensure complete vasodilation. The EKG showed diffuse 1.5 mm ST depression (see supplemental Table 3, which illustrates both the SPECT and ETT findings [2010–2018]). The ischemic changes first presented at the end of stage III of the modified Bruce protocol and were present up to 4 minutes in the recovery phase; they reversed to baseline after administration of aminophylline. Partial inferior wall scarring was noted; the LVEF was 54%. The Single-photon emission computed tomography ETT myocardial perfusion imaging study showed a mild intensity small-sized ischemic anterior wall defect extending from the mid to basal segments, a large size moderate intensity ischemic defect involving the anterolateral, inferolateral wall extending form the mid to the basal segments, and a small size mild to moderate intensity ischemic defect in the inferior wall. Compared to the prior test from 2010, the apical ischemia was no longer present, and the left ventricular wall hypokinesis was improved.

His medical records indicated persistent, recurring, chronic angina that would wax and wane in both severity and frequency, until progressing to unstable angina (UA) in 2012. As previously indicated, he has failed all first-and second-line pharmaceutical polypharmaceutical therapies. Most notably was the addition of ranolazine, which helped keep his chronic angina under moderate symptomatic control for several years. Ultimately, similar to other medications, ranolazine was modified for maximal impact and failed.

In late October of 2017, the patient sought evaluation for medicinal cannabis (MC). On initial visit, he presented with chronic chest pain due to UA that had not been totally relieved with long-term morphine treatment (morphine instant release [IR], 15 mg/8 h) and cardiac medications (rosuvastatin, amlodipine, clopidogrel, ranolazine, aspirin, carvedilol, lisinopril, and zolpidem; see table supplemental 2, for all medications and dosages [2009–2019]). His chest pain was 3/10 during evaluation but reported it may reach a level of 7/10 and would radiate to the left side of his chest. The patient says he was unable to walk long distances or perform mild/moderate physical activity. His symptoms were usually relieved by rest. He would, however, also experience frequent occurrences of angina unprovoked by physical activity or exertion, thus defining the presence of UA. He was started on MC at that time.

In late November 2017, the patient was seen for his first follow-up and presented with a decrease in the frequency of his chronic pain (CP). He stressed how the pain used to occur a few times/day and is now noticeably less often. He was also able to walk more than he previously was, but he still cannot do any strenuous activity. He was using a combination of high Δ^9^-tetrahydrocannabinol (THC) and low cannabidiol (CBD) strains (1:340 CBD:THC) (see table, supplemental 4, which displays the various medical cannabis strains used by our patient). It was discussed that he should be decarboxylating his MC and using it in edible form. The patient was able to reduce his morphine dosage from every 8 hours to once per day (q.d. morphine IR, 15 mg).

He returned in late December 2017 without any new significant relief compared to the previous month's progress. He would still get a feeling of tightness across his chest and need to sit down (q2/mo). It was recommended that he try a high CBD strain (1.2:1 CBD:THC) in combination with the previous THC strain via edible butter extraction bidaily (see table, supplemental 4, which displays the various medical cannabis strains used by our patient). Early February 2018, the patient reported that his pain was down from the old quality and character of sudden pain (even at rest), reported as a 6/10. Now described as a more consistent quality of dull pain rated at 3/10. He had been eating the recommended combo on toast 2 to 3 times/day (t.i.d.) and reports being happier during the day. He was still using morphine once daily (q.d. morphine IR, 15 mg). Overall, his chest discomfort and pressure were down 50%.

During his follow-up in the beginning of March 2018, he recounted now going days without pain. The patients’ CP was reduced from a few times a day to a few times a week but is dull and not sudden or debilitating like the past. It was discussed to stop the morphine if the MC is effectively controlling his CP. Later that week, the patient had a follow-up with his cardiologist, and they were both in agreement to continue using the MC if it is helping his UA and CAD symptoms, as well as reducing his dosage of morphine.

During his follow-up with cardiology, he was started on 2 new medications losartan and isosorbide mononitrate; the latter of which is known to mitigate reflex tachycardia and would be beneficial in a patient utilizing cannabis for congestive heart failure (CHF). It is also noted that he presented with edema but did not have jugular vein distention. He was referred for follow-up testing, and in April of 2018, the patient underwent another ETT (see supplemental Table 3, which illustrates both the SPECT and ETT findings [2010–2018]).

The EKG showed no evidence of exercise-induced arrhythmias (although a rare premature ventricualar contraction was present), and there were no signs of ischemia changes, a significant improvement (i.e., any ST changes were <1 mm and did not fulfill criteria for ischemia). Together, these findings mark improved functional capacity.

On his next primary care follow-up visit, in mid-May 2018, he had been off morphine for 6 days and controlling his symptoms using the recommended combo of high-dose CBD oil daily (t.d.) and THC butter 2 to 3 times daily (t.i.d.). He described the pain as no longer crippling and saw an increase in his daily functioning and overall activity, that is, he was able to walk more and perform more strenuous activity.

Early July 2018, he was completely off morphine (morphine IR, 15 mg/8 h) since April using the recommended combo of high-dose CBD oil and THC butter, as outlined above. His chest pain was significantly less frequent (2 times/day from >5 times/day before treatment) and less intense (now a dull pain and numbness that travels to the left arm and back, about 4/10). He reported that his last bad day was 6-weeks prior (mid-May 2018) and rated the pain on that occasion as a 9/10.

As of late March 2019, the patient reported only 2 episodes of chest pain since July 2018. The last one occurred in early March 2019 and was associated with a significantly emotional event. The patient is still opioid free, for 10 months, and uses a premixed combination of high-dose CBD hemp oil and a low dose of THC (15:1 CBD/THC) or the recommended extraction (1.2:1 CBD:THC), as outlined above. There have been no changes to his medications.

## 3 Discussion

The observed cardiovascular benefits of MC outlined here in this case support current trends in the literature, that is, cell culture,^[5]^ systematic reviews,^[6]^ receptor cloning, and agonist/antagonist studies.^[7–9]^ The case also supports the hypothesis that components of cannabis appear to be cardioprotective,^[4–9]^ operating via a vast distribution of the ECS and noncannabinoid secondary messenger systems.

Cannabis-induced acute MI (AMI), however, is a paradoxical feature of cannabis; occurring in a small percentage of users without a history of CAD or other appreciable factors.^[3,10–12]^ The leading hypothesis for AMI involves ST segment abnormalities and “hyper stimulation of vagal tone."^[3]^ Nevertheless, the mechanism, epidemiology, and prevalence remain elusive.

Starkly contrasting cannabis-induced AMI, emerging data explore a multifactorial role of MC in the treatment of UA and CAD via modulation of the ECS, CB_1_, and CB_2_. Altered expression of the CB_1_ and CB_2_ receptors has been demonstrated in the myocardium of mice, as well as in human cell culture, for models of cardiovascular disease, CHF, and ischemic insult.^[3–5]^

Administration of low-dose THC, however, protected the myocardium from ischemic damage.^[4]^ These findings were supported by lower systemic troponin and a reduced ischemic infarct.^[3]^ The myocardium of a healthy left ventricle is rich in CB_1_ and CB_2_ in nearly identical quantities. Meanwhile, cell culture studies have demonstrated that this ratio of CB_1_:CB_2_ is significantly altered following cardiovascular pathology.

In cells from patients with CHF, CB_1_ was slightly, but significantly, downregulated, whereas CB_2_ was upregulated.^[5]^ Cell culture studies from patients with MIs, and large ischemic insults, showed no change in CB_1_ and an upregulation of CB_2_.^[4,5]^ In addition, CB_1_ antagonism promotes cardiac remodeling following MI.^[5]^ Thus, the downregulation of CB_1_ observed in CHF might be maladaptive.

Recent studies have supported the role of CB_1_ and CB_2_, located systemically and in the cardiovascular system, as exhibiting both pathologic and protective effects depending on receptor agonism/antagonism^[3]^ with a strong correlation for CB_1_ inhibition and therapeutic benefits in cardiovascular pathology.^[4]^ The ECS has a functional role in coagulation and atherosclerosis.

Platelet cell membranes, and atherosclerotic plaques, have both CB_1_ and CB_2_.^[4]^ Low-dose oral THC inhibits atherosclerosis progression via pleiotropic immunomodulatory effects in apolipoprotein-E knockout models,^[9]^ as well as inhibiting lymphoid proliferation and macrophage chemotaxis, in a dose-dependent manner.^[4,9]^ This effect can also be inhibited by a CB_2_ antagonist.^[4]^ A similar function was observed for CB_2_ in vascular smooth muscle cells in coronary arteries. Depending on receptor activation, the ECS can promote destruction of plaques; a potential druggable target.

THC is a CB_1_ partial agonist, known to promote systemic vasodilation in addition to negative inotropy.^[4]^ CBD, however, has little affinity for CB_1_ or CB_2_. Instead, acting as a CB_1_ inverse agonist indirectly via GPCRs. CBD also acts on noncannabinoid targets, that is, as a GPR-55 antagonist and 5-HT_1A_ agonist. CB_1_ blockade is a proposed druggable target in cardiomyopathies because inhibition reverses negative inotropy.^[4]^ We hypothesize that the high CBD content in the patient's cannabis oil, in conjunction with his standard cardiac care, to be responsible for his improved functional capacity.

Several hypotheses outline the importance of upregulated CB_2_ in cardiomyocytes following cardiovascular insult. Most notably, CB_1_ and CB_2_ are hypothesized to play a dual role in regulating positive/negative inotrope through GPCRs. Specifically, the inhibitory Gi and Go proteins, and/or Gq proteins that resemble myocardial 5-HT_2A_.^[5]^ CB_2_ agonists are even known to be cardioprotective during times of acute ischemia, limiting myocardial damage during prolonged oxygen deprivation. An increase in CB_2_ might be responsible for the compensatory and beneficial mechanisms observed in CHF.^[5]^ All of which can be of considerable benefit for a heart, such our patients’, that never adequately revascularized.

## 4 Conclusion

This case report supports MC as an adjunctive treatment for UA and CAD. We hypothesize that our patient's improved metabolic exercise tests, UA, CP, reduced ischemic changes during stress tests, and overall increase in cardiovascular health/functional capacity, to be the result of a multifactorial mechanism. The mechanism of the ECS, its role in cardiovascular function, CP, and other systemic pathological states, have yet to be fully elucidated. It is, however, possibly the result of mixed CB_1_ and CB_2_ agonists/antagonists acting systemically on cardiomyocytes, vascular smooth muscle, and inflammatory cytokines/proinflammatory cells. Thus, increased appreciation of the involvement of the ECS in cardiac function and CP could result in greater use of agents targeting this system for CAD and related conditions.

## Acknowledgments

Gerald P. Tracy MD, Donald Haas MD, Stephanie D. Nichols PharmD, and Gabi N. Waite PhD provided feedback on earlier versions of this report. Iris Johnston tracked down all interlibrary loans. Lastly, we thank Dr Sonia Lobo PhD for generous contribution, as her department of research at GCSOM paid for the publication of this case report.

## Author contributions

**Conceptualization:** Brian L. Shaffer.

**Data curation:** Brian L. Shaffer.

**Formal analysis:** Brian V. Entler, Garrison M. Davis, Marc A. Incitti.

**Funding acquisition:** Brian V. Entler, Brian J. Piper.

**Investigation:** Brian V. Entler, Brian L. Shaffer.

**Methodology:** Brian V. Entler, Garrison M. Davis, Marc A. Incitti.

**Project administration:** Brian V. Entler, Brian J. Piper.

**Resources:** Brian J. Piper.

**Software:** Brian J. Piper.

**Supervision:** Brian V. Entler, Brian J. Piper.

**Writing – original draft:** Brian V. Entler.

**Writing – review & editing:** Brian V. Entler, Brian J. Piper.

## Supplementary Material

Supplemental Digital Content

## Supplementary Material

Supplemental Digital Content

## Supplementary Material

Supplemental Digital Content

## Supplementary Material

Supplemental Digital Content
