# Supplementary material for: Application of medical cannabis in unstable angina and coronary artery disease: A case report
Source: Medicine (Baltimore). 2021 Mar 19;100(11):e25172. doi: 10.1097/MD.0000000000025172 (PMC7982176; doi:10.1097/MD.0000000000025172)
Supplement: Supplemental Digital Content [file medi-100-e25172-s001.docx]

| **Supplemental Table 1 – Summary of Cardiac History (2009-2010).** | | |
| --- | --- | --- |
|  | **Procedure** | **Findings** |
| December, 2009 | CT Angiogram | Calcification in the LMCA and the proximal LAD; estimated at least a 50% luminal compromise. Disease in the RMCA was also noted. |
| January, 2010 | ECHO | Normal left ventricular systolic function. |
| January, 2010 | Cardiac Catheterization | The RMCA was small, non-dominant, with subtotal occlusion. There was 10% luminal compromise of the LMCA. Heavy calcification was noted within all of the diagonals of the LAD, while the LAD itself was difflusely diseased with areas of ectatic dilation and moderate stenosis, 60-70%. There was an aneurysmal dilation in the proximal LAD and a bare-metal stent was deployed distal to the dilation, in the mid-LAD. The left circumflex (LCX) was the dominant vessel and the AV segment was diffusely dilated and ectatic. The first and second obtuse marginal (OM1 and OM2) were 100% occluded and were noted to fill retrograde. A cutting balloon was unable to cross the area of disease in the both the OM1 and OM2. The OM3 had multiple stenotic regions with the worst being 90% occluded and a bare-metal stent was deployed at the location of the near-total occlusion. The posterior descending artery (PDA) was large and also diffusely alherosclerotic with multiple angiographic stenotic areas of varying severity; the worst located in the distal segments. |
| LMCA, left main coronary artery, LAD, left anterior descending artery. RMCA, right main coronary artery. | | |

**Supplemental Table 1 – Summary of Cardiac History (2009-2010).** Displaying the patients past cardiac medical history; specifically CT angiogram, ECHO, and cardiac catheterization findings.
