# Supplementary material for: Application of medical cannabis in unstable angina and coronary artery disease: A case report
Source: Medicine (Baltimore). 2021 Mar 19;100(11):e25172. doi: 10.1097/MD.0000000000025172 (PMC7982176; doi:10.1097/MD.0000000000025172)
Supplement: Supplemental Digital Content [file medi-100-e25172-s002.docx]

| **Supplemental Table 3 -** **Single-photon emission computerized tomography and Exercise Tolerance Test findings (2010-2018)**. | | | | | | | | |
| --- | --- | --- | --- | --- | --- | --- | --- | --- |
|  | **Speed (miles/hour)** | **Grade (%)** | **Achieved Maximum Predicted Heart Rate (MPHR)** | **Total Exercise Time (min)** | **Stage** | **METs (Metabolic exercise Test)** | **Procedures** | **Notable EKG Findings** |
| **Feb-10** | 2.5 mph | 12 | 96 | 5 | - | 7 | SPECT | 1.5mm ST depressions-horizontal to down sloping in the inferolateral leads. |
| **Sep-10** | 1.7 mph | 10 | 72 | 1 min 49 sec | 1 | 4.6 | ETT | 2 mm ST depression, exercise induced PVCs, and the test was abandoned. He did not complete stage I. |
| **Apr-11** | - | - | - | 2 | 3 | - | SPECT | Began to experience significant pain and had not achieved the target heart rate. The test was converted to a pharmacologic stress test and Regadenosine was administered t ensure complete vasodilation. |
| **Apr-11** | 1.7 mph | 10 | 78 | 10 | 3 | 4.6 | SPECT | 0.5 mm ST depression in leads II, III, aVF,V3,V4, V5, and V6. The ischemic changes presented at the end of stage land were present up to 4 mins into the recovery phase, they reversed to baseline after administration of aminophylline. |
| **Apr-18** | 1.8 mph | 15 | 70 | 4 min 41 sec | 2 | 6.1 | ETT | No evidence of exercise induced arrhythmias (although a rare PVC was present) and there were no signs of ischemia. |
| SPECT, Single-photon emission computerized tomography; ETT. [Exercise Tolerance Test; PVC, Pre-ventricular contraction](https://www.brighamandwomens.org/heart-and-vascular-center/procedures/standard-exercise-tolerance-test) | | | | | | | | |

**Supplemental Table 3 -** **Single-photon emission computerized tomography and Exercise Tolerance Test findings (2010-2018)**. The patient has an extensive history of SPECT myocardial perfusion studies demonstrating ischemia of moderate severity involving a large area of the anterolateral, as well as the inferolateral and inferior segments of myocardium. Comparison of the patients two exercise tolerance tests, which utilized a modified Bruce protocol, demonstrates mark improved functional capacity. Compared to 2011, his test in 2018 showed no evidence of exercise induced arrhythmias and there were no signs of ischemia.
