# Supplementary material for: Application of medical cannabis in unstable angina and coronary artery disease: A case report
Source: Medicine (Baltimore). 2021 Mar 19;100(11):e25172. doi: 10.1097/MD.0000000000025172 (PMC7982176; doi:10.1097/MD.0000000000025172)
Supplement: Supplemental Digital Content [file medi-100-e25172-s003.docx]

| **Supplemental Table 2- Medications and dosages (2009-2019).** | | | | |
| --- | --- | --- | --- | --- |
| Date | Medication | Dosage | Interval | Comments |
| 12/30/2009 |  |  |  | Patient Presents for Second Opinion |
|  | Atorvastatin | 40 mg | Daily | - |
|  | Metoprolol | 50 mg | 2x, Daily | - |
|  | Aspirin | 325 mg | Daily | - |
|  |  |  |  |  |
| 1/8/2010 |  |  |  | Echo/Cardiac Cath/Stents Placed |
|  | Aspirin | 325 mg | Daily | Dual Anti-lately therapy for 1 mo. Following stent placement. Must maintain Clopidogrel therapy for at least 9 mo. |
|  | Clopidogrel | 75 mg | Daily | Dual Anti-platelet therapy for 1 mo. Following stent placement. Must maintain Clopidogrel therapy for at least 9 mo. |
|  |  |  |  |  |
| 2/17/2010 |  |  |  | Nuclear Stress Test- Follow Up |
|  | Atorvastatin | 40 mg | Daily | Medication modified for maximal impact. Switched from Atorvastatin, which he failed, to Rosuvastatin. |
|  | Metoprolol | 50 mg | 2x, Daily |  |
|  | Aspirin | 325 mg | Daily | Now recommend for life-long treatment |
|  | Clopidogrel | 75 mg | Daily | Now recommend for life-long treatment |
|  | Rosuvastatin | 20 mg | Daily | Initiated |
|  | Niacin | 500 mg | 2x, Daily | Initiated |
|  | Omega-3 | 1000 mg | 2x, Daily | Initiated |
|  |  |  |  |  |
| 2/24/2010 |  |  |  |  |
|  | Carvedilol | 3.125 mg | Unknown | Initiated |
|  | Metoprolol | 50 mg | 2x, daily | Terminated |
|  |  |  |  |  |
| 3/17/2010 |  |  |  | ECHO/Doppler- Follow Up |
|  | Aspirin | 325 mg | Daily | Life-long treatment |
|  | Clopidogrel | 75 mg | Daily | Life-long treatment |
|  | Rosuvastatin | 20 mg | Daily | - |
|  | Niacin | 500 mg | 2x, Daily | - |
|  | Omega-3 | 1000 mg | 4x, Daily | Increased from twice, to four times. |
|  | Carvedilol | 6.25 mg | 1.5 Tabs, Daily | Carvedilol dosage increased |
|  | Lisinopril | 25 mg | Daily | Initiated |
|  |  |  |  |  |
| 5/26/2010 |  |  |  |  |
|  | Aspirin | 325 mg | Daily | Life-long treatment |
|  | Clopidogrel | 75 mg | Daily | Life-long treatment |
|  | Rosuvastatin | 20 mg | Daily | - |
|  | Niacin | 500 mg | 2x, Daily | Terminated due to adverse side effects |
|  | Omega-3 | 1000 mg | 4x, Daily | - |
|  | Carvedilol | 6.25 mg | 1.5 Tabs, Daily | - |
|  | Lisinopril | 25 mg | Daily | - |
|  |  |  |  |  |
| 9/15/2010 |  |  |  | ETT |
|  | Aspirin | 325 mg | Daily | Life-long treatment |
|  | Clopidogrel | 75 mg | Daily | Life-long treatment |
|  | Rosuvastatin | 20 mg | Daily | - |
|  | Omega-3 | 1000 mg | 4x, Daily | - |
|  | Carvedilol | 12.5 mg | 2x, Daily | Carvedilol dosage increased |
|  | Lisinopril | 25 mg | Daily | - |
|  |  |  |  |  |
| 10/5/2010 |  |  |  | ECHO/Doppler- Follow Up |
|  | Aspirin | 325 mg | Daily | Life-long treatment |
|  | Clopidogrel | 75 mg | Daily | Life-long treatment |
|  | Rosuvastatin | 20 mg | Daily | - |
|  | Omega-3 | 1000 mg | 4x, Daily | - |
|  | Carvedilol | 12.5 mg | 2x, Daily | - |
|  | Lisinopril | 25 mg | Daily | - |
|  |  |  |  |  |
| 1/17/2011 |  |  |  |  |
|  | Aspirin | 325 mg | Daily | Life-long treatment |
|  | Clopidogrel | 75 mg | Daily | Life-long treatment |
|  | Rosuvastatin | 40 mg | Daily | Rosuvastatin dosage increased |
|  | Omega-3 | 1000 mg | 4x, Daily | - |
|  | Carvedilol | 12.5 mg | 2x, Daily | - |
|  | Lisinopril | 25 mg | Daily | - |
|  |  |  |  |  |
| 3/16/2011 |  |  |  |  |
|  | Aspirin | 325 mg | Daily | Life-long treatment |
|  | Clopidogrel | 75 mg | Daily | Life-long treatment |
|  | Rosuvastatin | 40 mg | Daily | Rosuvastatin dosage decreased due to muscle aches and pains. |
|  | Omega-3 | 1000 mg | 4x, Daily | - |
|  | Carvedilol | 12.5 mg | 2x, Daily | - |
|  | Lisinopril | 25 mg | Daily | - |
|  |  |  |  |  |
| 5/6/2011 |  |  |  | Nuclear Stress Test- Follow Up |
|  | Aspirin | 325 mg | Daily | Life-long treatment |
|  | Clopidogrel | 75 mg | Daily | Life-long treatment |
|  | Rosuvastatin | 20 mg | Daily | - |
|  | Omega-3 | 1000 mg | 4x, Daily | - |
|  | Carvedilol | 12.5 mg | 2x, Daily | Carvedilol dosage increased. Additional 6.25 mg, taken at night. Continued 12.5 mg 2x, daily |
|  | Carvedilol | 6.25 mg | At night | Initiated |
|  | Lisinopril | 5 mg | Daily | Dosage decreased |
|  |  |  |  |  |
| 7/18/2011 |  |  |  | ECHO/Doppler- Follow Up |
|  | Aspirin | 81 mg | 2x, Daily | Dosage decreased, Life-long treatment |
|  | Clopidogrel | 75 mg | Daily | Life-long treatment |
|  | Rosuvastatin | 20 mg | Daily | - |
|  | Omega-3 | 1000 mg | 4x, Daily | - |
|  | Carvedilol | 12.5 mg | 2x, Daily | - |
|  | Carvedilol | 6.25 mg | At night | Terminated |
|  | Lisinopril | 5 mg | Daily | - |
|  | Ranolazine | 500 mg | Unknown | Initiated |
|  |  |  |  |  |
| 9/12/2011 |  |  |  |  |
|  | Aspirin | 81 mg | 2x, Daily | Life-long treatment |
|  | Clopidogrel | 75 mg | Daily | Life-long treatment |
|  | Rosuvastatin | 20 mg | Daily | - |
|  | Omega-3 | 1000 mg | 4x, Daily | - |
|  | Carvedilol | 12.5 mg | 2x, Daily | - |
|  | Lisinopril | 5 mg | Daily | - |
|  | Ranolazine | 500 mg | Unknown | - |
|  |  |  |  |  |
| 1/10/2012 |  |  |  |  |
|  | Aspirin | 81 mg | 2x, Daily | Life-long treatment |
|  | Clopidogrel | 75 mg | Daily | Life-long treatment |
|  | Rosuvastatin | 20 mg | Daily | - |
|  | Omega-3 | 1000 mg | 4x, Daily | - |
|  | Carvedilol | 12.5 mg | 2x, Daily | - |
|  | Lisinopril | 5 mg | Daily | - |
|  | Ranolazine | 500 mg | 2x, Daily | - |
|  |  |  |  |  |
| 5/4/2012 |  |  |  |  |
|  | Aspirin | 81 mg | 2x, Daily | Life-long treatment |
|  | Clopidogrel | 75 mg | Daily | Life-long treatment |
|  | Rosuvastatin | 20 mg | Daily | - |
|  | Omega-3 | 1000 mg | 4x, Daily | - |
|  | Carvedilol | 12.5 mg | 2x, Daily | - |
|  | Lisinopril | 5 mg | Daily | - |
|  | Ranolazine | 500 mg | 2x, Daily | - |
|  |  |  |  |  |
| 10/27/2017 |  |  |  | Initial Consultation for MC |
|  | Rosuvastatin | 20 mg | Daily | - |
|  | Amlodipine | 5 mg | Daily | Medication not seen in prior physician encounters, not a new prescription. |
|  | Clopidogrel | 75 mg | Daily | Life-long treatment |
|  | Ranolazine | 500 mg | 2x, Daily | - |
|  | Aspirin | 81 mg | 2x, Daily | Life-long treatment |
|  | Carvedilol | 12.5 mg | Daily | - |
|  | Lisinopril | 10 mg | Daily | Dosage increased compared to previous records. |
|  | Morphine | 15 mg | 2x, Daily | Medication not seen in prior physician encounters, not a new prescription either. |
|  | Zolpidem | 5 mg | Daily | - |
|  |  |  |  |  |
| 11/22/2017 |  |  |  | First Follow-Up- MC |
|  | Rosuvastatin | 20 mg | Daily | - |
|  | Amlodipine | 5 mg | Daily | - |
|  | Clopidogrel | 75 mg | Daily | Life-long treatment |
|  | Ranolazine | 500 mg | 2x, Daily | - |
|  | Aspirin | 81 mg | 2x, Daily | Life-long treatment |
|  | Carvedilol | 12.5 mg | 2x, Daily | - |
|  | Lisinopril | 10 mg | Daily | - |
|  | Morphine | 15 mg | Daily | - |
|  | Zolpidem | 5 mg | Daily | Terminated |
|  |  |  |  |  |
| 12/27/2017 |  |  |  | Second Follow-Up- MC |
|  | Rosuvastatin | 20 mg | Daily | - |
|  | Amlodipine | 5 mg | Daily | - |
|  | Clopidogrel | 75 mg | Daily | Life-long treatment |
|  | Ranolazine | 500 mg | 2x, Daily | - |
|  | Aspirin | 81 mg | 2x, Daily | Life-long treatment |
|  | Carvedilol | 12.5 mg | 2x, Daily | - |
|  | Lisinopril | 10 mg | Daily | - |
|  | Morphine | 15 mg | Daily | - |
|  |  |  |  |  |
| 2/6/2018 |  |  |  | Third Follow-Up- MC |
|  | Rosuvastatin | 20 mg | Daily | - |
|  | Amlodipine | 5 mg | Daily | - |
|  | Clopidogrel | 75 mg | Daily | - |
|  | Ranolazine | 500 mg | 2x, Daily | - |
|  | Aspirin | 81 mg | 2x, Daily | - |
|  | Carvedilol | 12.5 mg | 2x, Daily | - |
|  | Lisinopril | 10 mg | Daily | Terminated |
|  | Morphine | 15 mg | 1x, Daily | - |
|  |  |  |  |  |
| 3/6/2018 |  |  |  | Fourth Follow-Up- MC |
|  | Rosuvastatin | 20 mg | Daily | - |
|  | Amlodipine | 5 mg | Daily | - |
|  | Clopidogrel | 75 mg | Daily | Life-long treatment |
|  | Ranolazine | 500 mg | 2x, Daily | - |
|  | Aspirin | 81 mg | 2x, Daily | Life-long treatment |
|  | Carvedilol | 12.5 mg | 2x, Daily | - |
|  | Morphine | 15 mg | 1x, Daily | - |
|  |  |  |  |  |
| 3/16/2018 |  |  |  | First Follow-Up with Cardiology- Post-MC |
|  | Rosuvastatin | 20 mg | Daily | - |
|  | Amlodipine | 5 mg | Daily | - |
|  | Clopidogrel | 75 mg | Daily | Life-long treatment |
|  | Ranolazine | 500 mg | 2x, Daily | - |
|  | Aspirin | 81 mg | 2x, Daily | Life-long treatment |
|  | Carvedilol | 12.5 mg | 2x, Daily | - |
|  | Morphine | 15 mg | 1x, Daily | - |
|  | Losartan | 100 mg | Daily | Initiated |
|  | Isosorbide Mononitrate | 30 mg | Daily | Initiated |
|  | Montelukast | 10 mg | Daily | - |
|  |  |  |  |  |
| 5/17/2018 |  |  |  | Fifth Follow-Up with MC |
|  | Rosuvastatin | 20 mg | Daily | - |
|  | Amlodipine | 5 mg | Daily | - |
|  | Clopidogrel | 75 mg | Daily | Life-long treatment |
|  | Ranolazine | 500 mg | 2x, Daily | - |
|  | Aspirin | 81 mg | 2x, Daily | Life-long treatment |
|  | Carvedilol | 12.5 mg | 2x, Daily | - |
|  | Morphine | 15 mg | 1x, Daily | Terminated |
|  | Losartan | 100 mg | Daily | - |
|  | Isosorbide Mononitrate | 30 mg | Daily | - |
|  | Montelukast | 10 mg | Daily | - |
|  | Clonazepam | 0.5 mg | As needed | Initiated for morphine withdrawal symptoms |
|  |  |  |  |  |
| 7/17/2018 |  |  |  | Sixth Follow-Up with MC |
|  | Rosuvastatin | 20 mg | Daily | - |
|  | Amlodipine | 5 mg | Daily | - |
|  | Clopidogrel | 75 mg | Daily | Life-long treatment |
|  | Ranolazine | 500 mg | 2x, Daily | - |
|  | Aspirin | 81 mg | 2x, Daily | Life-long treatment |
|  | Carvedilol | 12.5 mg | 2x, Daily | - |
|  | Losartan | 100 mg | Daily | - |
|  | Isosorbide Mononitrate | 30 mg | Daily | - |
|  | Montelukast | 10 mg | Daily | - |
|  | Clonazepam | 0.5 mg | As needed |  |
| 3/26/2019 |  |  |  | Seventh follow-up with MC |
|  | Rosuvastatin | 20 mg | Daily | - |
|  | Amlodipine | 5 mg | Daily | - |
|  | Clopidogrel | 75 mg | Daily | Life-long treatment |
|  | Ranolazine | 500 mg | 2x, Daily | - |
|  | Aspirin | 81 mg | 2x, Daily | Life-long treatment |
|  | Carvedilol | 12.5 mg | 2x, Daily | - |
|  | Losartan | 100 mg | Daily | - |
|  | Isosorbide Mononitrate | 30 mg | Daily | - |
|  | Montelukast | 10 mg | Daily | - |
|  | Clonazepam | 0.5 mg | As needed | - |
| **Supplemental Table 2- Medications and dosages (2009-2019).** Due to the complex nature of the patient’s cardiac history (i.e. acute myocardial infarction, unstable angina, and congestive heart failure), he has an extensive history of poly-pharmaceutical treatment. Notably, he has a history of responding well to new medications when they are first introduced, producing significant results in his functional capacity. However, every first line mediation has been modified for maximal impact and failing. | | | | |
|  |  |  |  |  |
|  |  |  |  |  |
|  |  |  |  |  |
|  |  |  |  |  |
