# Supplementary material for: Application of medical cannabis in unstable angina and coronary artery disease: A case report
Source: Medicine (Baltimore). 2021 Mar 19;100(11):e25172. doi: 10.1097/MD.0000000000025172 (PMC7982176; doi:10.1097/MD.0000000000025172)
Supplement: Supplemental Digital Content [file medi-100-e25172-s004.docx]

**Supplemental Table 4**- **Medical Cannabis.**

| **Strain** | **THC**  **Concentration**  **(%)** | **CBD**  **Concentration**  **(%)** |
| --- | --- | --- |
| Blueberry #32 | 14 | 0.04 |
| Cannatonic | 6 | 7 |
| AK-47 | 18 | 0.05 |
| Kush VIII | 24 | 0.11 |
| Orange Bud | 17 | 0.05 |
| S.A.G.E #20 | 23 | 0.07 |
| THC, Tetrahydrocannabinol; CBD, Cannabidiol . | | |

**Supplemental Table 4**- **Medical Cannabis.** The patient initially tried a variety of medical cannabis strains, including numerous combinations. He did not experience much symptomatic relief with strains that contained a high dose of THC. Once the patient began to utilize the high CBD strains, however, he began to demonstrate the most benefit. All THC and CBD percentages reported were provided to the physician by the state licensed dispensary where the patient purchases his products.
